# Population pharmacokinetics of intravenous sufentanil in critically ill patients supported with extracorporeal membrane oxygenation therapy

**DOI:** 10.1186/s13054-019-2508-4

**Published:** 2019-07-09

**Authors:** Jongsung Hahn, Seungwon Yang, Kyoung Lok Min, Dasohm Kim, Byung Hak Jin, Changhun Park, Min Soo Park, Jin Wi, Min Jung Chang

**Affiliations:** 1 Department of Pharmacy and Yonsei Institute of Pharmaceutical Sciences, College of Pharmacy, Yonsei University, Incheon, 21983 Republic of Korea; 2 Department of Pharmaceutical Medicine and Regulatory Sciences, Colleges of Medicine and Pharmacy, Yonsei University, Incheon, 21983 Republic of Korea; 3 Department of Clinical Pharmacology, Severance Hospital, Yonsei University College of Medicine, Seoul, 03722 Republic of Korea; 4 Department of Pediatrics, Yonsei University College of Medicine, Seoul, 03722 Republic of Korea; 5 Division of Cardiology, Department of Internal Medicine, Gachon University Gil Medical Center, 21 Namdong-daero 774beon-gil, Namdong-gu, Incheon, 21565 Republic of Korea; 6 Division of Cardiology, Department of Internal Medicine, Yonsei University College of Medicine, 50 Yonsei-Ro, Seodaemun-Gu, Seoul, 03722 Republic of Korea

**Keywords:** Anaesthetics, intravenous, Sufentanil, Extracorporeal membrane oxygenation, Pharmacokinetics, Temperature

## Abstract

**Background:**

Sufentanil is commonly used for analgesia and sedation during extracorporeal membrane oxygenation (ECMO). Both ECMO and the pathophysiological changes derived from critical illness have significant effects on the pharmacokinetics (PK) of drugs, yet reports of ECMO and sufentanil PK are scarce. Here, we aimed to develop a population PK model of sufentanil in ECMO patients and to suggest dosing recommendations.

**Methods:**

This prospective cohort PK study included 20 patients who received sufentanil during venoarterial ECMO (VA-ECMO). Blood samples were collected for 96 h during infusion and 72 h after cessation of sufentanil. A population PK model was developed using nonlinear mixed effects modelling. Monte Carlo simulations were performed using the final PK parameters with two typical doses.

**Results:**

A two-compartment model best described the PK of sufentanil. In our final model, increased volume of distribution and decreased values for clearance were reported compared with previous PK data from non-ECMO patients. Covariate analysis showed that body temperature and total plasma protein level correlated positively with systemic clearance (CL) and peripheral volume of distribution (V2), respectively, and improved the model. The parameter estimates of the final model were as follows: CL = 37.8 × EXP (0.207 × (temperature − 36.9)) L h^−1^, central volume of distribution (V1) = 229 L, V2 = 1640 × (total plasma protein/4.5)^2.46^ L, and intercompartmental clearance (*Q*) = 41 L h^−1^. Based on Monte Carlo simulation results, an infusion of 17.5 μg h^−1^ seems to reach target sufentanil concentration (0.3–0.6 μg L^−1^) in most ECMO patients except hypothermic patients (33 °C). In hypothermic patients, over-sedation, which could induce respiratory depression, needs to be monitored especially when their total plasma protein level is low.

**Conclusions:**

This is the first report on a population PK model of sufentanil in ECMO patients. Our results suggest that close monitoring of the body temperature and total plasma protein level is crucial in ECMO patients who receive sufentanil to provide effective analgesia and sedation and promote recovery.

**Trial registration:**

Clinicaltrials.gov NCT02581280, December 1st, 2014.

## Background

Venoarterial extracorporeal membrane oxygenation (VA-ECMO) is a temporary mechanical circulatory support for patients with cardiac failure [1, 2]. Because ECMO is invasive, analgesia and sedation are important to limit responsiveness, prevent accidental decannulation, and maintain ECMO flows, all of which promote recovery [3–5]. The use of opioids is standard practice during ECMO [6–8].

Sufentanil is a synthetic opioid drug, which has a rapid onset and is 5–10 times more potent than fentanyl [9]. It is highly protein bound (91–93%) [10], metabolised by the liver, and excreted as metabolites in the urine (2% unchanged, 80% metabolites) [11]. A large variability in sufentanil pharmacokinetics (PK) is expected in ECMO patients due to the combination of ECMO, drug characteristics, and disease factors [12]. Volume of distribution (Vd) is altered owing to physiologic changes related to critical illness, hemodilution, and sequestration in ECMO circuit, while clearance (CL) is variable owing to organ dysfunction and non-pulsatile flow in VA-ECMO [13–15]. ECMO could act as a reservoir that prolongs the effect of sedatives even after the drugs have been discontinued [16]. Despite the widespread use of ECMO, the literature regarding sufentanil PK and ECMO was based on only in vitro analysis, which showed 83% loss of sufentanil in ECMO circuits at 24 h [17]. In the present study, we aimed to develop a population PK model of sufentanil in ECMO patients and identify covariates associated with sufentanil exposure in order to suggest a more rational dosing recommendation.

## Methods

### Study design and ethics approval

This was a prospective, cohort study conducted at the cardiac intensive care unit in Severance Cardiovascular Hospital, a university-affiliated tertiary care hospital in Seoul, Republic of Korea, between January 2016 and June 2017. The study was approved by the Institutional Review Board (IRB No.: 4-2014-0919) of Severance Hospital and was registered at Clinicaltrials.gov (NCT02581280). Written informed consent was obtained from the patients or the legal surrogates of unconscious patients. This study complied with the Strengthening the Reporting of Observational studies in Epidemiology (STROBE).

### Study population

Twenty patients aged 19 years or older, who received sufentanil-based analgesia and sedation during VA-ECMO, were enrolled in this study. The exclusion criteria were younger than 19 years, known allergy to sufentanil, and taking any medication that could cause potential drug-drug interactions or alter sufentanil concentrations.

### Dosing, administration, and data collection

ECMO patients received sufentanil for maintenance of analgesia and sedation supplemented as needed with midazolam. Sufentanil dosing in our centre was based on patients’ body weight, with initial infusion doses of 12.5 (< 60 kg) or 17.5 μg h^−1^ (≥60 kg). An initial bolus of 3 (< 60 kg) or 5 mg (≥ 60 kg) midazolam was given, with an initial infusion dose of 4.5 mg h^−1^. Management of pain should be guided by routine pain assessment of Nonverbal Pain Scale. Nurses assessed the depth of sedation, indicating the prevalent Richmond Agitation Sedation Scale (RASS) in their work shift. Analgesia and sedation protocol was to keep patients deeply sedated during the first few days of ECMO followed by intermediate or light sedation before ECMO discontinuation when possible. The infusion rates of sufentanil and midazolam were modified to achieve a target RASS score, and each dose adjustment was recorded.

Data on demographics, organ function, ECMO, vital signs, and drug dosing were collected from the electronic medical records.

### Extracorporeal membrane oxygenation

The ECMO circuit included a centrifugal blood pump with a pump controller (Capiox® SP-101, Terumo Inc., Tokyo, Japan), an air-oxygen mixer (Sechrist® Ind., Anaheim, CA, USA), and conduit tubing (Capiox® EBS Circuit with X coating, Terumo Inc.). The days on ECMO, ECMO flow rate, and ECMO pump speed were recorded.

### Sample collection and plasma concentration assay

The study was initiated during the first 48 h of starting ECMO. Blood samples were collected after 3 and 12 h of infusion and then every 24 h until 96 h had elapsed. When infusion was discontinued for any reason, blood was sampled after 0, 0.5, 1, 2, 6, and 12 h, and then every 24 h until 72 h had elapsed. Each blood sample (2 mL) was drawn from an existing arterial line and collected in a tube containing ethylenediaminetetraacetic acid as an anticoagulant. The blood samples were centrifuged at 1500×*g* for 10 min at 4 °C, and the plasma was immediately stored at − 80 °C until needed.

The plasma concentrations of sufentanil were analysed using a validated HPLC system (Agilent Technologies, CA, USA) coupled with a 4000 Qtrap liquid chromatograph-mass spectrometer (ASICX, Concord, Ontario, Canada). The plasma samples were denatured with acetonitrile containing 0.5 μg mL^−1^ prazosin as an internal standard. The mixture was vortexed and centrifuged at 150,000×*g* for 10 min at 4 °C. HPLC was performed on a Kinetex C_18_ analytical column (4.6 × 50 mm; particle size 2.6 μm; Phenomenex, Torrance, CA, USA) with a mobile phase consisting of 0.1% formic acid in acetonitrile at a flow rate of 0.055 mL min^−1^. The lower limit of quantification for sufentanil was 0.02 μg L^−1^. The assay was validated between 0.02 and 10 μg L^−1^ with inter- and intra-assay coefficients of variation of < 15%.

### Population PK model development

The population PK model was developed using a first-order conditional estimation method with an interaction (FOCE+I) algorithm in the nonlinear mixed effects modelling software NONMEM® version 7.4 (ICON Development, Ellicott City, MD, USA). Pirana® ver. 2.9.2 and Xpose® ver. 4.0 (http://xpose.sourceforge.net) in R® ver. 3.2.4 (http://www.r-project.org) were used to visualise and evaluate the models. One-, two-, and three-compartment models were evaluated as the structural PK models. Inter-individual variability (IIV) for the PK parameters was modelled assuming a log-normal distribution: *θ*_*i*_ = *θ*_Pop_ × EXP(*η*_*i*_), where *θ*_*i*_ is the individual value of the parameter *θ* in the *i*th individual, *θ*_Pop_ is the population value of this parameter, and *η*_*i*_ is a random variable with mean zero and variance *ω*_*η*_^2^ [18]. Proportional models for residual variability was used: *c*_*ij*_ = *cp*_*ij*_ × (1 + *ε*_*ij*_) in which *c*_*ij*_ is the *j*th observed concentration of the *i*th individual, *cp*_*ij*_ is the corresponding predicted concentration, and *ε*_*ij*_ is a random variable with mean zero and variance *σ*^2^.

The likelihood ratio test was used to evaluate statistical significance between nested models where a decrease in the objective function value (OFV), a statistical equivalent to the − 2 log likelihood of the model, of at least 3.84 was considered statistically significant for an added parameter (*χ*^2^ distribution, degrees of freedom (df) = 1, *p* < 0.05). In addition, bias of the goodness-of-fit plots (observed versus population predicted concentrations, observed versus individual predicted concentrations, conditional weighted residuals (CWRES) versus population predicted concentrations, and CWRES versus time after dosing), visual improvement of individual plots, confidence intervals of parameter estimates, and shrinkage were assessed. The aim of this study was to examine the potential effect of various covariates of the model structural parameters. The following covariates were investigated: sex, age, weight, lean body weight, body mass index, tympanic body temperature, total plasma protein, partial pressure of carbon dioxide, plasma pH, estimated glomerular filtration rate, serum creatinine, total bilirubin, alanine transaminase, aspartate transaminase, use of continuous renal replacement therapy, ECMO pump speed, and ECMO flow rate. The estimated parameters were plotted against each covariate to identify its influence. Continuous covariates (Cov) were incorporated into the structural model with centering on their median values within the population and tested using power (1), linear (2), and exponential (3) equations:

1 $$ {\theta}_{\mathrm{Pop}}={\theta}_{\mathrm{TV}}\times {\left\{\mathrm{Cov}/\mathrm{Median}\left(\mathrm{Cov}\right)\right\}}^{\theta_{\mathrm{Cov}}} $$

2 $$ {\theta}_{\mathrm{Pop}}={\theta}_{\mathrm{TV}}+{\theta}_{\mathrm{Cov}}\times \left\{\mathrm{Cov}-\mathrm{Median}\left(\mathrm{Cov}\right)\right\} $$

3 $$ {\theta}_{\mathrm{Pop}}={\theta}_{\mathrm{TV}}\times \mathrm{EXP}\left({\theta}_{\mathrm{Cov}}\times \mathrm{Cov}/\mathrm{Median}\left(\mathrm{Cov}\right)\right) $$

where *θ*_TV_ is the typical value of the parameter and *θ*_Cov_ quantifies the covariate effect. The covariate model building was carried out in a stepwise process. In the forward selection, a *P* value of < 0.05 was used (a decrease in OFV of at least 3.84, df = 1), while in the backward elimination, a *P* value of < 0.01 was applied (a decrease in the OFV of at least 6.64, df = 1).

### Model evaluation and simulations

To evaluate stability in the final model, a non-stratified bootstrap analysis was performed using the PsN Toolkit [19]. A bootstrap with 5000 runs was performed on the final model to evaluate the internal validity of the parameter estimates and their corresponding 95% confidence intervals (CIs). The model performance was evaluated by means of prediction-corrected visual predictive checks (pc-VPCs). One thousand datasets were simulated from the final model, and the median and 90% CI of the simulated data were plotted along with the observed concentrations. To illustrate the effect of body temperature and total plasma protein level on predicted sufentanil concentrations, Monte Carlo simulations were performed using PK parameters from the final model. We assumed that sufentanil was continuously infused for 120 h with a rate of 12.5 or 17.5 μg h^−1^, which were the doses most frequently used in our ECMO patients. The median parameter values for the patient population were obtained with five different levels of body temperature (33, 35, 36.7, 38 and 39 °C) and four different total plasma protein levels (2, 4, 6, and 8 g dL^−1^) by simulating 1000 individuals in each case.

## Results

### Patients

Twenty ECMO patients were included, with a median age of 55 years, median weight of 69.4 kg, and median APACHE II score of 29 at the initiation of ECMO support. All the patients received mechanical ventilation and started ECMO during the first 12 h after the onset of myocardial infarction (MI). The median duration of VA-ECMO and sufentanil infusion was 138 and 110 h, respectively. Nine patients concurrently received continuous venovenous hemodiafiltration (CVVHDF) during ECMO (Table 1). Desired sedation level was reached or nearly reached (observed RASS = target RASS ± 1) in all patients. A population PK analysis was conducted with 106 plasma samples from the 20 patients. Concentration records that were below the lower limit of quantification were excluded from the analysis.

**Table 1:** Characteristics of the study patients

	Number/median (range) (*n* = 20)
Age (years)	55 (23–88)
Male	16
Body weight (kg)	69.4 (52.9–92.5)
Lean body weight (kg)	55.3 (36.8–58.6)
Body mass index (kg m^−2^)	24.7 (20.5–31.8)
Indication for VA-ECMO	
Acute myocardial infarction	4
ST-elevation myocardial infarction	12
Non-ST-elevation myocardial infarction	4
APACHE II	29 (15–36)
Time between onset of MI and initiation of ECMO (h)	2 (0.5–12)
Blood chemistry, serum levels	
Total plasma protein (g dL^−1^)	4.5 (2.1–6)
Total bilirubin (mg dL^−1^)	1.9 (0.3–6.6)
Blood urea nitrogen (mg dL^−1^)	21.3 (7.5–58)
Serum creatinine (mg dL^−1^)	1.4 (0.4–4.9)
Partial pressure of carbon dioxide (mmHg)	29.1 (13.5–46.7)
Tympanic body temperature (°C)	36.9 (33–38.7)
Use of continuous venovenous hemodiafiltration	9
Duration of VA-ECMO (h)	138 (52.9–263)
ECMO flow rate (L min^−1^)	3 (0.6–4.1)
Duration of sufentanil infusion (h)	110 (34–260)
Sufentanil infusion rate	
17.5 μg h^−1^	15
12.5 μg h^−1^	5

### Population PK model building

A two-compartment model parameterised in terms of systemic clearance (CL), central volume of distribution (V1), peripheral volume of distribution (V2), and intercompartmental clearance (*Q*) was preferred to a one- and three-compartment model. Residual variability was described with a proportional residual error model. IIVs were included for CL and V2, since they significantly improved model performance. The structural model had an OFV of − 343.1. In the univariate covariate analysis, body temperature and total bilirubin were identified as significant covariates of CL, resulting in a drop in OFV of − 9.1 and − 6.0 points, respectively. In addition, total protein and lean body weight were significant covariate candidates of V2 with *∆* OFVs of − 9.3 and − 3.4 points, respectively. After the forward selection and backward elimination, total bilirubin for CL and lean body weight for V2 were removed.

Thus, the final PK model is described as follows:

- CL = 37.8 × EXP (0.207 × (temperature − 36.9)) L h^−1^
- V1 = 229 L
- V2 = 1640 × (total plasma protein/4.5)^2.46^ L
- *Q* = 41 L h^−1^

### Model evaluation and simulation

The final parameter estimates and IIVs along with their bootstrap CIs are provided in Table 2. All parameters had acceptable relative standard error values. The population mean estimates were contained within the 95% CIs of the bootstrap results.

**Table 2:** Parameter estimates and bootstrap confidence intervals

Parameter	Structural model (RSE%) [shrinkage%]	Final model		
		Final model (RSE%) [shrinkage%]	Bootstrap (5000 replicates)	
			Median	95% CI (2.5–97.5%)
Fixed effects				
**Θ**_***CL***_	39.5 (11)	37.8 (3)	37.4	27.0, 47.8
**Θ**_***V*1**_	220 (23)	229 (10)	223	66.2, 352
**Θ**_***V*2**_	1620 (26)	1640 (9)	1629	755, 4304
**Θ**_***Q***_	35.8 (17)	41 (12)	42.1	21.5, 67.6
**Θ**_***Temp***_	–	0.207 (5)	0.21	0.066, 0.437
**Θ**_***T.Prot***_	–	2.46 (7)	2.51	0.892, 5.12
Random effects				
Inter-individual variability				
***ω***_***CL***_^**2**^	0.196 (46) [15]	0.167 (57) [20]	0.197	0.03, 0.484
***ω***_***V*2**_^**2**^	1.41 (35) [21]	1.13 (48) [22]	0.968	0.081, 2.08
Residual variability				
***σ***^**2**^_***proportional***_	0.0915 (19) [10]	0.0841 (5) [9]	0.0743	0.047, 0.116

*RSE%*, relative standard error; RSE% = (standard error/parameter estimate) × 100; *CL*, systemic clearance; *V1*, central volume of distribution; *V2*, peripheral volume of distribution; *Q*, intercompartmental clearance

**Fig. 1:**
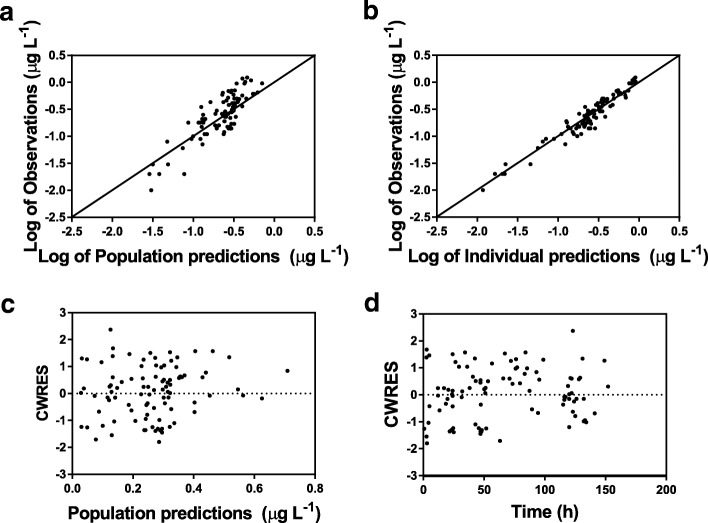
Goodness-of-fit plots of the final population pharmacokinetic model. Log of observed sufentanil concentrations versus log of population predicted concentrations (**a**) and versus log of individual predicted concentrations (**b**), and conditional weighted residuals (CWRES) versus population predicted concentrations (**c**) and versus time (**d**)

**Fig. 2:**
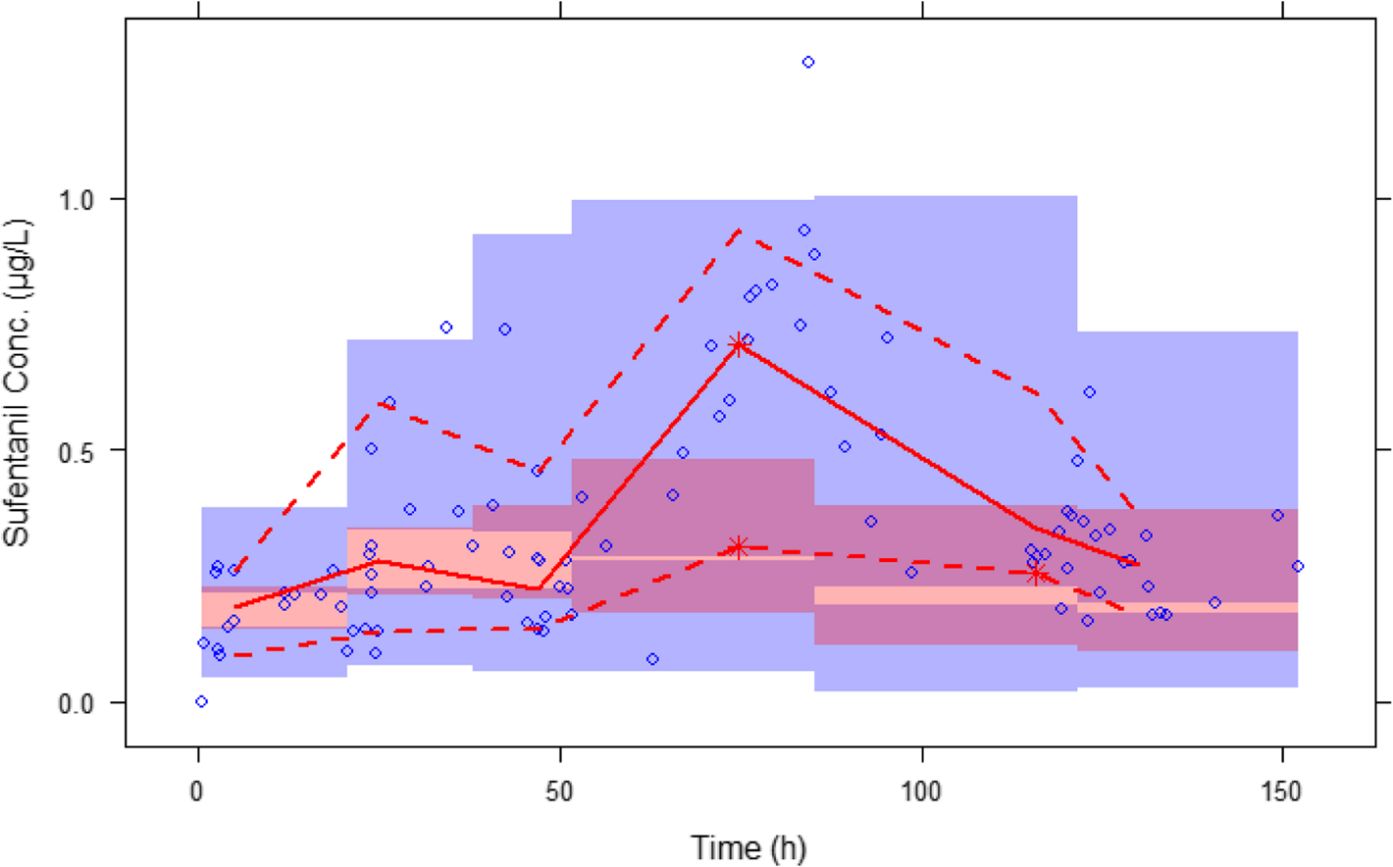
Prediction-corrected visual predictive checks (pc-VPCs) of the final population pharmacokinetic model. Open circles, observed sufentanil concentrations; solid line, median; lower and upper dashed lines, 5th and 95th percentiles of the simulated data, respectively; shaded areas, 95% confidence intervals for simulated predicted median, 5th percentile, and 95th percentile constructed from 1000 simulated datasets of individuals from the original dataset

**Fig. 3:**
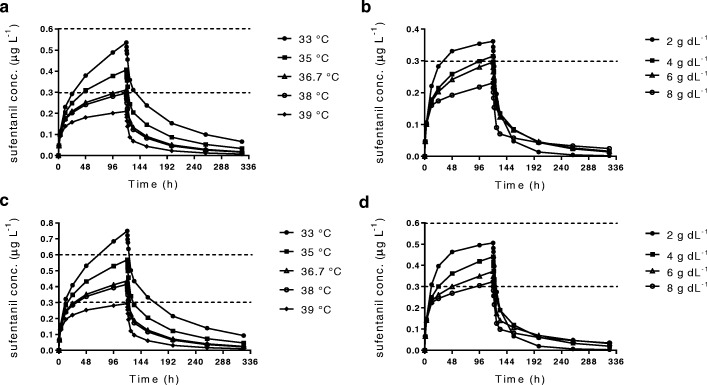
Simulated mean sufentanil concentrations for 120-h sufentanil infusion in patients. Patients were stratified for body temperature (**a**, **c**) or total plasma protein (**b**, **d**). **a** 12.5 μg h^−1^ infusion in patients with total plasma protein levels of 4.5 g dL^−1^. **b** 12.5 μg h^−1^ infusion in patients with body temperatures of 36.9 °C. **c** 17.5 μg h^−1^ infusion in patients with total plasma protein levels of 4.5 g dL^−1^. **d** 17.5 μg h^−1^ infusion in patients with body temperatures of 36.9 °C

Figure 1 a–d show the goodness-of-fit plots. Both the population predictions and individual predictions were distributed relatively symmetrically around the line of unity when plotted against the observations. In addition, the CWRES versus time and versus population predictions did not show any trends and were approximately uniformly distributed around zero in the final model. The pc-VPCs revealed that the 5th to 95th percentiles of the predicted data overlaid most of the observed data, indicating good precision of the PK model (Fig. 2). The final covariate model was then used for simulations. Figure 3 shows the Monte Carlo simulated sufentanil concentrations during 120-h infusion using two different dosing regimens, stratified by the body temperature and total plasma protein level. The target concentrations were set to 0.3–0.6 μg L^−1^. Overall, the concentrations of sufentanil are increased in patients with low body temperature and low total plasma protein levels.

Figure 3 a and c reveal the effect of different body temperatures on sufentanil concentrations. With a dose of 17.5 μg h^−1^, the concentrations of sufentanil from 24 to 120 h were within the target concentrations in patients with a body temperature of 35–38 °C, whereas they were above the target concentrations in patients developing hypothermia (33 °C) and under the target concentrations in patients with high fever (39 °C).

Figure 3b and d show the effect of different total plasma protein levels on sufentanil concentrations. With a dose of 17.5 μg h^−1^, sufentanil concentrations were within target concentrations in most patients, whereas a dose of 12.5 μg h^−1^ was low for patients with total plasma protein levels of 4–8 g dL^−1^.

## Discussion

We analysed a population of 20 critically ill ECMO patients who received sufentanil-based analgesia and sedation, and we described a population PK model. A two-compartment model with first-order elimination fitted the time course of the total plasma sufentanil concentrations best. In our final model, increased Vd (V1, 229 L; V2, 1640 L, standardised total plasma protein level of 4.5 g dL^−1^) and decreased values for clearance (CL, 37.8 L h^−1^, standardised temperature of 36.9 °C; *Q*, 41 L h^−1^) were reported compared with previous PK data from non-critically ill patients (V1, 37.1 L; V2, 92.7 L; CL, 76.2 L h^−1^; *Q*, 52.2 L h^−1^) [20] and critically ill patients not undergoing ECMO (Vd, 1582 L; CL, 56 L h^−1^) [21]. Sufentanil, as a highly lipophilic (logP = 3.24) and high protein-binding (91–93%) drug [22], could be largely sequestered in the ECMO circuit, which mimics an increase in Vd [15]. Moreover, a systemic inflammatory response, which can be triggered by the patient’s clinical condition or the initiation of ECMO, alters permeability of the blood-brain barrier and impacts the Vd of sufentanil [13]. Decreased CL may have resulted from the reduced hepatic blood flow and impaired hepatic function in critically ill patients [23]. Although nine patients received CVVHDF concomitantly with ECMO, it would have little effect on sufentanil PK. Primary mechanism of sufentanil clearance is the liver. Also, the drug is lipophilic, exhibits highly protein binding, and has a relatively high molecular weight (386.552 g/mol). Thus, it is expected that sufentanil would not be removed by CVVHDF, with limited clearance by VA-ECMO.

Body temperature and total protein level were found to be significant covariates of sufentanil PK, and interestingly, weight-related covariates were not included in the final model. In previous sufentanil PK studies in patients undergoing coronary artery bypass surgery, adding weight as a covariate showed neither a significant change in log-likelihood nor an improvement in predictive ability due to the large impact of coronary artery bypass surgery on sufentanil PK [16, 24]. Since ECMO also has a large impact on sufentanil PK, we concluded that body weight is rendered insignificant as a factor in our final model.

The relationship between body temperature and sufentanil systemic clearance was described as follows: CL = 37.8 × EXP (0.207 × (temperature − 36.9)) L h^−1^. These results are in agreement with the findings of previous studies, in which sufentanil showed decreased clearance in hypothermic patients [25–27]. There are several processes that may be responsible for a decrease in sufentanil CL as body temperature drops. Sufentanil, a drug with a high liver-extraction ratio (hepatic extraction ratio of 0.7), is expected to be sensitive to blood flow alterations [11]. When body temperature drops, total hepatic blood flow is assumed to be markedly reduced [28], which then reduces the hepatic elimination of sufentanil. Furthermore, sufentanil metabolism occurs mainly via the cytochrome P450 system (CYP450), which is known to be strongly affected by temperature. Low temperature changes the binding pocket confirmation of CYP3A4, which reduces substrate affinity for CYP3A4 binding sites and slows CYP3A4 metabolic activity [29]. In recent studies, CYP3A4*1G genetic polymorphism was found to be correlated with a lower amount of sufentanil consumption due to impaired activity of CYP3A4 [30, 31]. The frequency of the CYP3A4*1G variant allele showed big difference by ethnicity, which was 0.188–0.279 in Chinese patients [32] and 0.079 in Caucasian patients [33]. In further studies, CYP3A4 polymorphism should be considered when extrapolating our data to other patient groups.

The effect of temperature is especially relevant in ECMO patients who show variability in body temperature for many reasons. The body temperature of ECMO patients could drop because of repeated blood transfusion, infusion of fluid, severe infection, and sepsis. In addition, to minimise brain damage, the body temperatures of ECMO patients after cardiac arrest are not allowed to exceed 36 °C over 24 h. In contrast, some ECMO patients could develop fever, which is associated, for example, with inflammation, elevated sympathetic tone, and catheter-related infections.

We also found that total plasma protein level was correlated positively with V2. Our results are different from those of other studies, in which total protein level was negatively correlated with V2 [34–36]. One teicoplanin PK study demonstrated that a reduction in protein binding due to hypoproteinaemia could promote the distribution of the free form of teicoplanin into extravascular or intracellular spaces, thus increasing the volume of distribution [35]. However, our finding could be explained by the fact that sufentanil binding is affected mainly by the plasma concentration of α1-acid glycoprotein, and not by the total plasma protein level. Low total protein levels might reflect impaired hepatic function, which could produce low apparent volumes of distribution [37]. We want to highlight that our results are observatory and further studies are needed to fully uncover the relationship between total protein level and volume of distribution. Furthermore, our estimates of V2 shrinkage were relatively high (33%), so these estimates should be interpreted with caution.

Targeted sufentanil plasma concentrations in critically ill patients have not yet been determined accurately with PK/pharmacodynamic studies. Pain and sedation management are important consideration in the care of the ECMO patients, and no practice guidelines exist for this population. Recent evidences suggest sedative/analgesic protocols aiming for minimal and lighter sedation to improve clinical outcomes [38, 39]. With limited data in patients with cardiac surgery together [40–43], we suggested a target concentration between 0.3 and 0.6 μg mL^−1^ to ensure sedation and better clinical outcomes. Overall, an infusion of 17.5 μg h^−1^ seems better than 12.5 μg h^−1^ in most ECMO patients, except hypothermia patients (33 °C). In hypothermic patients, over-sedation, which could induce respiratory depression, needs to be monitored especially when their total plasma protein level is low. With assessment of the analgesia and sedative levels, dose reductions should be considered. On the contrary, optimal levels of analgesia and sedation could not be induced with commonly used doses in hyperthermic patients, which suggests that an increased dose should be considered.

Our study did have several limitations. First, although the hepatic clearance of sufentanil is largely dependent on hepatic plasma flow, we did not observe hepatic blood flow as a potential covariate of CL in our PK model. Second, the concomitant use of sedating and paralysing medications prevented us from exploring the pharmacodynamics of sufentanil in terms of the level of sedation and analgesia. Future prospective studies that control for the presence of concomitant sedating and paralysing agents and that measure the exact degree of sedation and analgesia score are needed to link drug concentrations to the level of sedation and analgesia to determine appropriate concentrations. Nevertheless, the model developed in our study could be used for future sufentanil dosing considerations and the design of clinical studies in patients using ECMO.

## Conclusions

In conclusion, to the best of our knowledge, this is the first report on a population PK model of sufentanil in ECMO patients. The sufentanil volume of distribution was increased, and clearance was decreased in VA-ECMO patients compared with the values from previously reported non-ECMO patients. Body temperature and total plasma protein level correlated positively with CL and V2, respectively. The influence of body temperature and total plasma protein on the PK of sufentanil should be considered. Further research should focus on the pharmacodynamics of sufentanil, such as sedation and analgesia levels and haemodynamic stability in patients during VA-ECMO.

## Data Availability

The datasets generated and/or analysed during the current study are not publicly available due to privacy concerns and institutional policy, but are available from the corresponding author on reasonable request.

## Abbreviations

CIs: Confidence intervals
CL: Systemic clearance
CRRT: Continuous renal replacement therapy
CVVHDF: Continuous venovenous hemodiafiltration
CWRES: Conditional weighted residuals
CYP450: Cytochrome P450 system
df: Degrees of freedom
ECMO: Extracorporeal membrane oxygenation
EDTA: Ethylenediaminetetraacetate
IIV: Inter-individual variability
IPRED: Individual predictions
NONMEM: Nonlinear mixed effects modelling
OFV: Objective function value
pc-VPC: Prediction-corrected visual predictive checks
PK: Pharmacokinetics
PRED: Population predictions
Q: Intercompartmental clearance
V1: Central volume of distribution
V2: Peripheral volume of distribution
VA-ECMO: Venoarterial ECMO
